# Blood handling and leukocyte isolation methods impact the global transcriptome of immune cells

**DOI:** 10.1186/s12865-018-0268-6

**Published:** 2018-10-30

**Authors:** Brittany A. Goods, Jacqueline M. Vahey, Arthur S. Steinschneider, Michael H. Askenase, Lauren Sansing, J. Christopher Love

**Affiliations:** 1 Department of Biological Engineering, Koch Institute for Integrative Cancer Research at the Massachusetts Institute of Technology, Cambridge, MA 02139 USA; 2 Department of Electrical Engineering and Computer Science, Massachusetts Institute of Technology, Cambridge, MA 02139 USA; 3 Department of Neurology, Yale School of Medicine, New Haven, CT 06520 USA; 4 Department of Chemical Engineering, Koch Institute for Integrative Cancer Research at the Massachusetts Institute of Technology, Cambridge, MA 02139 USA; 5 The Broad Institute of the Massachusetts Institute of Technology and Harvard, Cambridge, MA 02142 USA

**Keywords:** Immune profiling, Peripheral blood mononuclear cells, Transcriptome, RNA-seq

## Abstract

**Background:**

Transcriptional profiling with ultra-low input methods can yield valuable insights into disease, particularly when applied to the study of immune cells using RNA-sequencing. The advent of these methods has allowed for their use in profiling cells collected in clinical trials and other studies that involve the coordination of human-derived material. To date, few studies have sought to quantify what effects that collection and handling of this material can have on resulting data.

**Results:**

We characterized the global effects of blood handling, methods for leukocyte isolation, and preservation media on low numbers of immune cells isolated from blood. We found overall that storage/shipping temperature of blood prior to leukocyte isolation and sorting led to global changes in both CD8^+^ T cells and monocytes, including alterations in immune-related gene sets. We found that the use of a leukocyte filtration system minimized these alterations and we applied this method to generate high-quality transcriptional data from sorted immune cells isolated from the blood of intracerebral hemorrhage patients and matched healthy controls.

**Conclusions:**

Our data underscore the necessity of processing samples with comparably defined protocols prior to transcriptional profiling and demonstrate that a filtration method can be applied to quickly isolate immune cells of interest while minimizing transcriptional bias.

**Electronic supplementary material:**

The online version of this article (10.1186/s12865-018-0268-6) contains supplementary material, which is available to authorized users.

## Background

Transcriptional profiling can yield valuable insights into cellular states and phenotypes across tissues and diseases [1, 2]. This approach is especially useful when applied to study cells and tissues in the context of perturbations such as drugs or disease state. These data allow for the inference of target pathways of interest [3], unique functional states [4], correlates of disease state [5], and novel subtype classifications [6].

Sequencing methods such as Smart-Seq2 to assess the transcriptome using RNA isolated from very low numbers of cells (~ 1–1,000) has enabled profiling of clinical samples containing very low numbers of cells, rare cell types, or rely on previously biobanked materials [7]. Many studies have identified optimal processing procedures and analysis methods for low-input or degraded RNA samples [8–11]. The availability of preservatives and stabilizing reagents for whole blood has also allowed the generation of high-quality data from ultra-low volumes of blood [12]. The application of transcriptional profiling to clinical samples, such as tissues and sorted populations of cells, however, presents unique and significant challenges associated with sample acquisition, sample processing, and tissue isolation. For example, multi-center studies often require centralized processing of patient samples; this transfer risks introducing bias. Additionally, RNA quality can vary widely between tissues [13], which can confound study design. Simply relying on acquiring enough high-quality samples to reach target cohort sizes with high RNA quality is not always practical with rare or difficult to procure samples.

Few previous studies have sought to identify artifacts introduced into transcriptional data by sample handling in the context of RNA-sequencing. For expression profiles generated by microarrays or quantitative PCR, studies have identified several factors that can affect resulting data. One such study found that incubation time prior to standard procedures for leukocyte collection can rapidly change blood transcriptomics [14]. Shipping at 4 °C may mitigate these changes, but does not completely abrogate the effects. Another study, using microarrays, investigated the effect of time, temperature, and preservation on the transcriptome of isolated peripheral blood mononuclear cells (PBMCs) [15]: each preservation material tested had an effect on the transcriptome, but relied on large volumes of blood. To date, there has been a paucity of studies that have characterized artifacts introduced by the method of leukocyte isolation method followed by low input RNA-sequencing.

Here, we characterized the global effects of blood handling, method of leukocyte isolation, and preservation on low numbers of immune cells isolated from blood. We found overall that the storage/shipping temperature of blood prior to leukocyte isolation and sorting led to global changes in both CD8^+^ T cells and monocytes, including alterations in immune-related gene sets. We found these alterations could be minimized through the use of a leukocyte filtration system, and we applied this method to generate high-quality transcriptional data from sorted immune cells isolated from the blood of patients with acute intracerebral hemorrhage and matched healthy controls. Our data demonstrate the utility of our filtration approach and underscore the necessity of processing samples with defined and closely matched protocols prior to transcriptional profiling.

## Results

### Simulated approach for sample handling

**Fig. 1:**
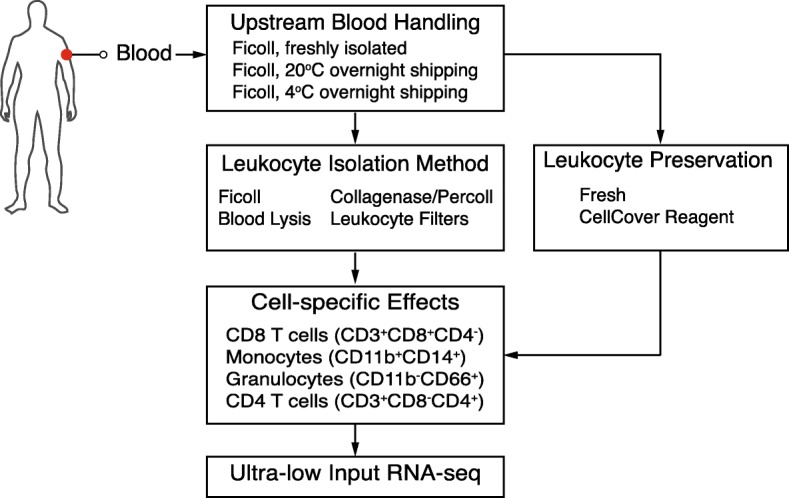
Simulated sample handling methods. Diagram outlines methods used to simulate various sample handling techniques representative of those encountered in clinical immunology, including: blood shipping temperature (20 °C or 4 °C), peripheral blood mononuclear cell isolation method (Ficoll gradient, collagenase plus percoll gradient, whole blood lysis, leukocyte filtration), and immune cell types of interest (CD8^+^ T cells, CD4^+^ T cells, granulocytes or monocytes)

We first sought to determine how methods for sample handling impact transcriptional data quality, composition, and downstream biological interpretation. We used blood since it is a readily available biological material that is often used for biomarker discovery and transcriptional profiling. To this end, we simulated several sample handling conditions that are typically encountered when profiling immune cells, and emphasized methods and handling techniques often associated with clinical trials or multi-site studies that require sample transport prior to analysis or tissue analysis (Fig. 1). Handling conditions included the effect of overnight shipping, various methods of leukocyte isolation, and the effect of a transcriptome preservation medium called CellCover. First, we compared processing over a Ficoll gradient directly after collection (freshly isolated) to whole blood shipped overnight at 20 °C or 4 °C (1 day at 20 °C and 1 day at 4 °C, respectively). Second, we tested the effect of the method used to isolate leukocytes from blood samples. Methods tested included i) Ficoll gradient isolation since this is typically used for blood (Ficoll), ii) collagenase/DNase digestion and subsequent isolation by percoll gradient since this is typically used for tissue (collagenase plus percoll), iii) RBC lysis as this is typically used for small amounts of aspirate or tissue (whole blood lysis), and iv) a modified method for leukocyte filtration (whole blood filtration). The filtration method used was an adaptation of the LeukoLock filter system where whole blood is applied to the filter, which captures leukocytes while allowing red blood cells and platelets to pass through. The filters are then washed to further remove red blood cells and platelets and leukocytes are subsequently isolated by back flushing the filter. The comparison of Ficoll to collagenase plus percoll or whole blood lysis was particularly important, since many studies aim to compare blood and tissue compartments. Finally, we wanted to quantify the effect of CellCover, a solution that stabilizes RNA and other biomolecules in live cells and tissue by inhibiting enzymatic hydrolysis. This media was added after leukocyte isolation and has the advantage of allowing for antibody and live cell staining. After leukocyte isolation for each condition, cells were stained (Additional file 1: Table S1) and analyzed by flow cytometry.

### Effects of blood shipping temperature, leukocyte isolation method, and preservation on percentage of cell types isolated

**Fig. 2:**
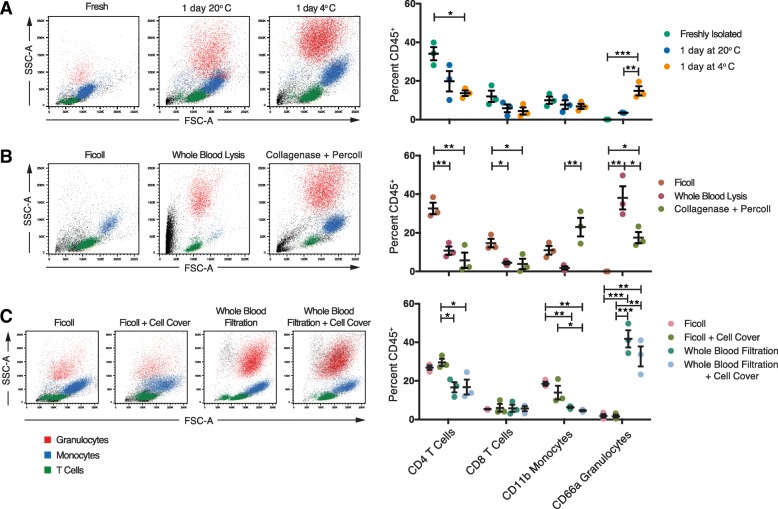
Sample handling has differential effects on the percent of immune cell types isolated. Blood was processed according to the sample simulation schematic for (**a**) blood handling (fresh, 1 day at 4 °C, or 1 day at room temperature (20 °C) followed by Ficoll isolation), (**b**) PBMC isolation method (Ficoll, whole blood lysis, or collagenase plus percoll gradient), or (**c**) PBMC shipping method (fresh versus CellCover reagent) for Ficoll or filtration isolation methods. For each, example plots on the left are colored according to T cells (CD4^+^, green), monocytes (CD11b^+^CD66a^−^, blue), granulocytes (CD11^+^CD66a^+^, red), or debris (black). Quantification of percentages of total CD45^+^ PBMCs are shown for each replicate (*n* = 3) on the right. Statistical analyses were performed by one-way ANOVA with Tukey’s multiple comparisons test within each cell type. Summary significance is shown in each panel and exact *P* values are reported in Additional file 1: Table S2

First we wanted to determine how storage/shipping, isolation, and preservation (Fig. 1) affected the percentage of immune cell types isolated. For each donor, we examined the percentage of the following immune cell types across each condition (Additional file 1: Figure S1): monocytes (CD11b^+^CD66^−^), granulocytes (CD11b^−^CD66^+^), CD4^+^ T cells (CD3^+^CD4^+^CD8^−^), and CD8^+^ T cells (CD3^+^CD4^−^CD8^+^). We found that shipping temperature, isolation method, and preservation all had some effect on the percentages of immune cell types isolated (Fig. 2), with some methods having a more significant impact than others (Additional file 1: Table S2). Globally, we found that shipping at 4 °C resulted in a statistically significant higher percentage of live granulocytes but a lower percentage of CD4^+^ T cells isolated (Fig. 2a). Shipping temperature did not significantly affect CD8^+^ T cells or monocytes. We also found that both whole blood lysis and the collagenase plus percoll gradient resulted in more debris overall; the latter resulted in a significantly higher yield of monocytes compared to whole blood lysis (Fig. 2b). Overall, both whole blood lysis and collagenase plus percoll methods resulted in a lower yield of CD4^+^ and CD8^+^ T cells. Finally, we found that the addition of the CellCover reagent did not significantly impact either ficoll or whole blood-filtered protocols (Fig. 2c). Additionally, we found that whole blood filtration led to significantly greater yields of granulocytes with less debris, while still enabling isolation of comparable frequencies of T cells and monocytes. Together, these data suggest that the method chosen for upstream cell isolation could have a large impact on the percentages of desired cell types, which will significantly impact the isolation of rare populations of cells.

### Blood handling and conventional leukocyte isolation methods alter the global transcriptome of monocytes and CD8^+^ T cells

Given that immune cells are poised to quickly react to their surroundings, we sought to determine how each sample handling condition could affect the global transcriptome of isolated immune cells. We sorted two populations of immune cells representative of the T cell (CD8^+^ T cells CD3^+^CD8^+^) and the innate (monocytes, CD11b^+^CD66a^−^) immune compartments into lysis buffer for low-input RNA-sequencing. RNA-sequencing libraries were generated as previously described [16]. In total, we profiled three healthy donors for each condition, resulting in 64 total libraries that were sequenced to a depth greater than 10 million reads (Additional file 2: Table S3). We found that the quality of libraries generated was not significantly affected by incubation temperature processing method, or preservation method, but that whole blood filtration resulted in slightly higher quality libraries for both T cells and monocytes (Additional file 1: Figure S2).

**Fig. 3:**
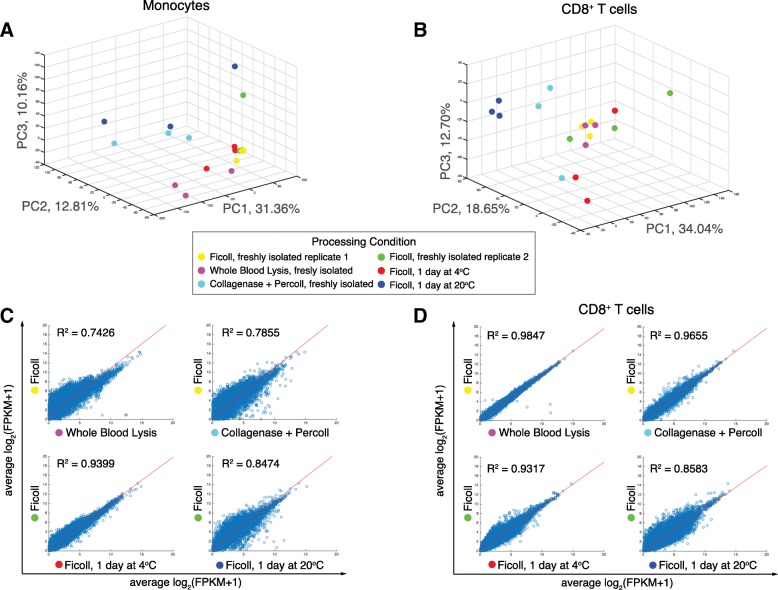
Effect of blood shipping and PBMC isolation methods on the global transcriptome of monocytes and T cells. CD8 T cells (CD3^+^CD8^+^) and Monocotyes (CD11b^+^CD66a^−^) were isolated according to each condition and profiled by RNA-sequencing. **a** Principal components analysis (PCA) of the resulting transcriptomes (log_2_(FPKM+ 1) > 0.1) across all handling conditions. **b** Pairwise-scatter plots of the average log_2_(FPKM+ 1) were fit using linear regression and R^2^ values are shown

To determine global effects of upstream handling and processing on the transcriptome, we performed principal component analysis (PCA) on all coding genes across each condition for monocytes (Fig. 3a) and CD8^+^ T cells (Fig. 3b) and are showing data projected along principal components 1 and 2 (PC1 and PC2). We also plotted pair-wise scatter plots of the average transcriptome (Fig. 3c and d) and each individual transcriptome (Additional file 1: Figures. S3 and S4) for each condition and performed linear regression. We found that for both monocytes and CD8^+^ T cells, the fresh ficoll-isolated conditions clustered closely (Fig. 3 a, b), suggesting good correlation between independent experiments. Unsurprisingly, we found that for both monocytes and CD8^+^ T cells, shipping at 20 °C resulted in transcriptomes that differed the most from the freshly-obtained Ficoll controls (Fig. 3b, d). We also found that collagenase plus percoll and whole blood lysis isolation methods had a large effect on the monocytes, whereas shipping at 4 °C resembled the freshly-obtained controls (Fig. 3a). Pair-wise scatter plots across all donors (Additional file 1: Figure S3) also showed that collagenase plus percoll and whole blood lysis methods led to induced alterations in biological reproducibility as compared to Ficoll controls for the monocytes. For the CD8^+^ T cells, the collagenase plus percoll and whole blood lysis methods did not have as large of an effect, with correlations remaining high across biological replicates (Additional file 1: Figure S4A) and on average (Fig. 3d). Overall, our data suggests that for monocytes, isolation with collagenase plus percoll or through whole blood lysis, and shipping at room temperature prior to isolation can induce global changes in the resulting transcriptome. For CD8^+^ T cells, our data suggests that shipping at room temperature induced the largest global transcriptional changes.

**Fig. 4:**
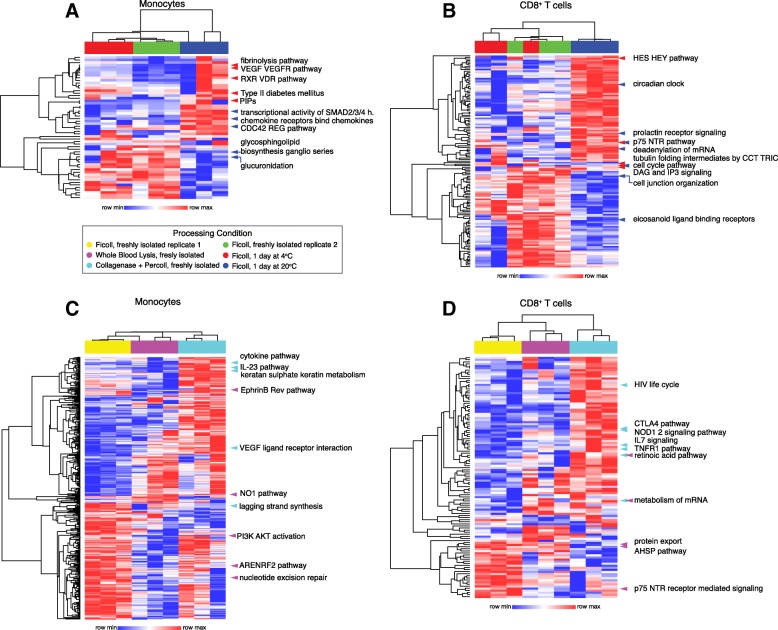
Isolation and shipping methods induce differential changes in enriched gene sets identified in CD8^+^ T cells and monocytes. Single sample gene set enrichment analysis (ssGSEA) was performed on all samples indicated by processing condition (color scale bars at top of each heatmap). Enrichment scores identified as significant between pairwise conditions were merged and hierarchically clustered across rows and columns. The top five significant gene sets for each comparison are highlighted with colored arrows, where the color indicates significance as compared to Ficoll control: Ficoll versus 4 °C in red and Ficoll versus 20 °C in blue for monocytes (**a**) and CD8^+^ T cells (**b**), and for Ficoll versus lysed in pink and Ficoll versus percoll in cyan for monocytes (**c**) and CD8^+^ T cells (**d**). In A, PIPs is short for synthesis of PIPs at the late endosomal membrane

In order to determine the biological nature of detected alterations, we performed single sample gene set enrichment analysis (ssGSEA) [17]. This method allows for comparison of enriched gene sets across all conditions of interest by generating individual enrichment scores for each gene set. Significantly different gene sets (*p* < 0.05) were identified through pair-wise comparisons of each condition tested to the freshly-isolated leukocytes obtained by Ficoll gradient as control for each processing condition (Additional file 3: Table S4). Gene sets that were significant for any comparison were merged and enrichment scores were hierarchically clustered across rows and columns, with gene sets as rows and samples as columns (Fig. 4). The top five significant gene sets are highlighted in each clustergram, where the color of each arrow indicates from which comparison it derives its significance to allow for global comparison. Overall, we found that each processing condition clustered together, indicating that sample processing alone can drive sample clustering. We also found that processing condition altered many gene sets, including immune-related gene sets. In general, temperature induced fewer changes than the method of leukocyte isolation. For monocytes, there was a cluster of gene sets that included Vascular endothelial growth factor (VEGF)-related signaling and RXR VDR pathway (retinoic acid related), that were significantly different between the shipping at 4 °C and fresh Ficoll conditions (Fig. 4a). This result may indicate that shipping at 4 °C could alter monocyte migration pathways and homeostasis pathways. For CD8^+^ T cells, shipping at 20 °C induced alterations in cell cycle (circadian clock), ecosanoid ligand binding, and prolactin receptor signaling, all of which contribute to immune homeostasis (Fig. 4b) [18]. We also found that leukocyte isolation method induced alterations in immune-cell related signaling pathways. For monocytes, isolation by collagenase plus percoll led to increased enrichment in cytokine pathways, IL-23, and VEGF receptor interaction (Fig. 4c). Conversely, isolation by whole blood lysis or collagenase plus percoll actually abrogated a distinct set of gene sets enriched in the Ficoll control, including Phosphatidylinositol-4,5-bisphosphate 3-kinase/protein kinase B (PI3K/AKT) signaling, lagging strand synthesis, and nitric oxide 1 (NO1) signaling, the latter of which plays a key role in monocyte migration [19]. For CD8^+^ T cells, isolation with collagenase plus percoll led to enrichment of a distinct set of gene sets, including those that are relevant to T cell functionality, including cytotoxic-T-lymphocyte-associated protein 4 (CTLA-4) pathway, IL-7 signaling, and TNF Receptor Superfamily Member 1A (TNFR1) pathway (Fig. 4d). The effect of whole blood lysis on T cells is less clear, but there was a cluster of enriched gene sets abrogated in this condition, which included neurotrophin receptor p75 (p75 NTR) signaling. Taken together, our data suggest that each isolation method induced specific alterations in gene signatures that may impact biological interpretation.

### Mechanical isolation by filtration has minimal effect on the global transcriptome of CD8^+^ T cells and monocytes

**Fig. 5:**
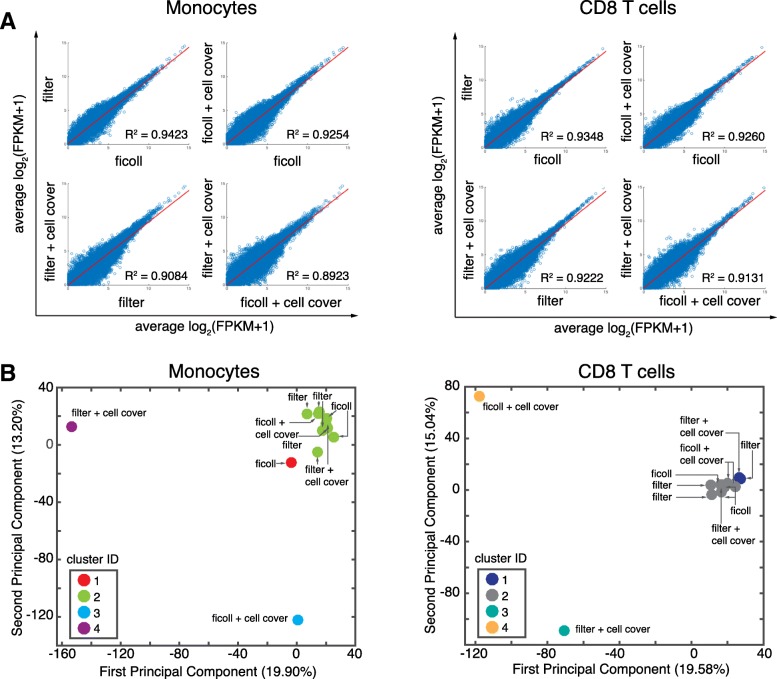
Effect of mechanical isolation and preservation on the global transcriptome of monocytes and CD8 T cells. Blood was processed according to the sample simulation schematic for mechanical filtration and preservation method (CellCover). CD8^+^ T cells (CD3^+^CD8^+^) and monocotyes (CD11b^+^CD66a^−^) were isolated and profiled by RNA-sequencing. **a** Pairwise-scatter plots of the average log_2_(FPKM+ 1) for each condition were fit using linear regression and R^2^ values are shown. **b** Principal components analysis (PCA) of the resulting transcriptomes (log_2_(FPKM+ 1) > 0.1) across all indicated conditions. Data were hierarchically clustered and the resulting cluster ID is shown on PCA plots

Given that we found striking differences induced by conventional methods for sample handling, we sought to determine the effect of a mechanical isolation (filtration) method on the global transcriptome. We also sought to determine if preservation with CellCover, a transcriptome preservation reagent, could minimize effects of temporal delays during sample processing. As above, we generated pairwise scatter plots of the average across replicates for each comparison (Fig. 5a) and performed PCA (Fig. 5b). We found that mechanical isolation by whole blood filtration had no significant effect on the quality, and in some cases actually improved the quality metrics of the resulting library (Additional file 1: Figure S2C, Table S1). We also found that the global transcriptome for both CD8^+^ T cells and monocytes remained similar when isolated with filters, with high correlation values to the Ficoll control (Fig. 5a).

The inclusion of CellCover may actually induce variability in both monocytes and CD8^+^ T cells. We observed clear outliers, regardless of filtration or Ficoll processing (Fig. 5b). We also performed ssGSEA and found overall fewer total gene sets altered (Additional file 4: Table S5). Of the top gene sets that were altered, surprisingly there were few related directly to immune function (Additional file 1: Figure S5A). For monocytes, unlike previous methods, we did not find alterations in VEGF or migration pathways, but did find maintained alterations in P75NTR signaling (Additional file 1: Figure S5A). For CD8^+^ T cells, we did not see induction of cytokine-related pathways, but did see alterations in pathways related to the cell matrix and platelets (Additional file 1: Figure S5B). Taken together, these data suggest that mechanical filtration, without CellCover, results in consistent transcriptomes generated by RNA-seq for both monocytes and CD8^+^ T cells.

### Application of leukocyte filtration to patient samples retains unique cell-type biology

**Fig. 6:**
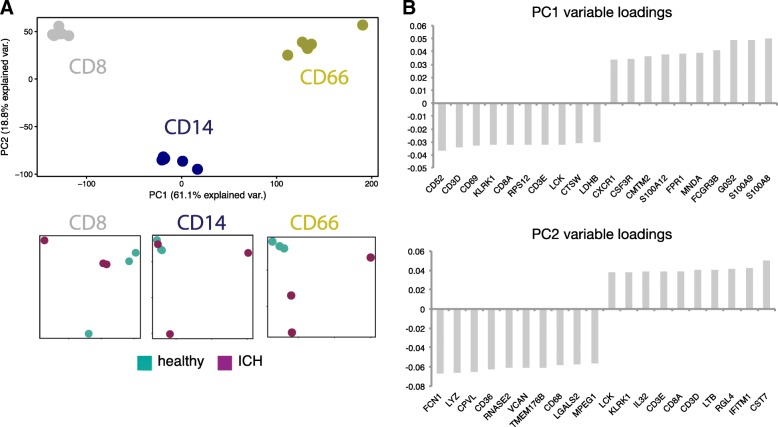
Leukocyte isolation by filtration of low-input samples maintains cell-specific and disease-specific signatures. Monocytes, granulocytes, and CD8^+^ T cells were isolated post PBMC filtration from the blood of intracerebral hemorrhage (ICH) patients and matched healthy donors (healthy). **a** PCA was performed on resulting transcriptomes and colored according to cell type (CD8, CD14, or CD66) or disease state (healthy or ICH). **b** Top 20 genes contributing to PC1 and PC2 are shown ordered by loading

Given that we found leukocyte filtration had minimal effect on the global transcriptome and allowed for fast processing of low volumes of biological material, we applied this method to low volumes of blood from intracerebral hemorrhage patients (ICH) and matched healthy donors (HD) to mimic material and workflows that would be obtained as part of a clinical study. We isolated leukocytes by filtration, stained for flow cytometry, and isolated monocytes, CD8^+^ T cells and granulocytes (Additional file 1: Figure S6). Each isolated subset yielded high-quality data by RNA-sequencing (Additional file 1: Figure S7), including granulocytes. PCA across all data showed that cell types clustered together, and within each cluster separation between ICH and healthy-derived subsets were evident (Fig. 6a). Notably, CD8^+^ T cells and CD66^+^ granulocytes primarily segregated along PC1, with little separation along PC2; CD14^+^ monocytes were distinct from these populations along both PC1 and PC2. We next wanted to look at the highest-ranking genes driving separation between cell types (Fig. 6b). We found several genes that drive separation along PC1 that are characteristic of CD8^+^ T cell identity (*Cd3d, Cd3e, Lck, CD8a*) or granulocyte identity (*S100a8*, *S100a9, Fpr1, Cxcr1*), agreeing with the observed separation of cells and granulocytes along this principal component. High-ranking genes driving separation along PC2 included several important for monocyte function (*Lyz, CD36, CD68*), in agreement with the separation of monocytes from other cell types along this principal component. Taken together, our data suggest that leukocyte filtration retains cell specific biology in clinical samples while providing a fast workflow that is compatible with low volumes of material.

## Discussion

Few studies have characterized artifacts that can be introduced into transcriptional data, especially data generated by sensitive methods like low-input RNA-sequencing. We sought to quantify how sample handling and leukocyte isolation method affects the global transcriptome of isolated immune cells, with emphasis on conditions that may be encountered when profiling blood and tissue (ie: solid tissue) associated with clinical trials and multi-center studies with the goal of providing data and guidance for early stage experimental design. We simulated upstream blood handling conditions (fresh, shipment of whole blood at 20 °C, or 4 °C overnight, where all were compared to fresh as a control), leukocyte isolation methods (Ficoll gradient isolation, collagenase/DNase digestion and subsequent Percoll gradient isolation, whole blood lysis, or a modified leukocyte filtration method, where Ficoll gradient isolation was used as a control), and leukocyte preservation methods (CellCover, where the absence of CellCover was used as a control).

We found that shipping temperature of blood prior to leukocyte isolation and sorting led to unique global changes in both CD8^+^ T cells and monocytes. These included biologically meaningful alterations in immune-related gene sets, such as monocyte migration and homeostasis pathways in monocytes at 4 °C and cell cycle pathways at 20 °C in T cells. Our data also show that each method for leukocyte isolation can significantly impact the percent of isolated immune cells. This outcome can have a large impact on transcriptional studies, especially when starting from low-input material, including small volume blood draws, tissue biopsies, or aspirates. Thus, it is critical when designing clinical studies to choose a method that maximizes the cell type of scientific interest for the study. For example, if the main interest is to isolate granulocytes, processing with whole blood lysis or the newly described filtration method would yield the greatest fraction of granulocytes from a sample. Conversely, if the main interest were T cells, isolation with a Ficoll gradient from a fresh sample would yield the greatest fraction of T cells in the immune compartment.

We also observed global alterations in the transcriptome due to leukocyte isolation method, suggesting that each handling and leukocyte isolation condition induces distinct alterations in the global transcriptome of monocytes and CD8^+^ T cells. Isolation by collagenase plus percoll led to alterations in cytokine pathways in monocytes and T cell signaling, like the CTLA-4 pathway, in T cells. The latter is crucial because inhibitory pathways are biologically meaningful in many contexts, including cancer [20]. Transcriptome alterations are unique to the immune cell type of interest, with little overlap in altered gene sets between conditions, suggesting cell-type specific effects. Previous studies have identified alterations in gene expression induced by a temporal delay in processing, and have shown that hypoxia and apoptosis signatures are induced [15]. Other studies have also found that delays in processing induce alterations in RNA-splicing in liquid biopsies across large cohorts, including those derived from public repositories [14]. Here, we found that differences in processing can induce significant alterations in immune-related signatures that could lead to misinterpretation of resulting data sets. To account for this effect, our data suggests that samples must be processed comparably, especially when comparing blood and tissue-derived immune cells.

Finally, we applied a novel leukocyte filtration method to study the transcriptomes of three immune cell populations in the context of health and ICH. We found that leukocyte filtration leads to high-quality global transcriptomes across different immune cell types, results in retained disease-specific information, and suggests that cell identities are preserved in transcriptional space through enrichment in well-studied immune cell gene signatures. Importantly, transcriptomes generated using this filtration approach were highly correlated with those derived from Ficoll isolation, allowing comparison to previous studies that have used Ficoll. The filtration method, however, additionally allows for the collection of high-quality transcriptional data from granulocytes, even after overnight shipping at 4 °C. Together with our data from healthy donors, this suggests that processing samples with filtration allows for the recovery of high quality transcriptomes from a broad range of leukocytes and preserves biologically meaningful contexts of the original samples.

## Conclusion

We found that the shipping temperature of blood and the method of isolation have global effects on the transcriptome of both immune cell types we studied. Specifically, our data suggests that isolation of immune cells from blood with collagenase plus percoll or through whole blood lysis, and shipping at room temperature prior to isolation can induce meaningful global alterations in monocyte transcriptomes. Similarly, for CD8^+^ T cells, shipping blood at room temperature prior to isolation induced the greatest global changes. These findings suggest that care should be taken when designing studies to ensure samples, both blood and tissue, are processing comparably. Strikingly, we found these alterations could be minimized through the use of a system for leukocyte filtration. We applied this method to generate high-quality transcriptional data from sorted immune cells isolated from the blood of ICH patients and healthy donors. Taken together, our data suggest that sample processing has drastic effects on resulting transcriptional data across immune cell types, and the immune-related effects of these methods may be mitigated through filtration.

## Methods

### Human subjects

A total of nine subjects were analyzed for cell sorting and transcriptional profiling by RNA-seq according to IRB approved protocols. A total of six subjects were healthy blood donors and three were intracerebral hemorrhage patients.

### Reagents

Lithium Heparin (catalog # 367886) and ACD-A (catalog # 364606) were obtained from BD Biosciences. Erythrocyte hypotonic lysis buffer was prepared by mixing 8.6 g NH4Cl in 1 L MilliQ water, and 0.2 um filtered. Azide-free FACS buffer was prepared with a final concentration of 2% BSA *w*/*v* and 1 mM EDTA. PBS (catalog # 14190144) was obtained from Gibco. BD Pharm Lyse (catalog # 555899) was purchased from BD Biosciences. Ficoll Paque PLUS (catalog # 17–1440-03) and Percoll (catalog # 17–0891-01) was obtained from GE Healthcare. Collagenase/displace (catalog # 10269638001) and DNAse (catalog # 10104159001) were obtained from Roche. The EasySep Human CD3 Positive Selection Kit II (catalog # 17851) was obtained from STEMCELL technologies. The monoclonal antibodies used to identify leukocyte subpopulations for cell sorting are listed in Additional file 1: Table S1.

### Shipping conditions

In order to test the effect of sample transport temperature, two commercially available shipping container systems for biological samples were utilized: 4 °C in an activated cold shipping container (medium size, standard duration, nanoCool©) and 20 °C in a controlled room temperature shipping container with two phase change material insulators (Saf-T-Temp® CRT 10/30 (2) in a STP-302, Saf-T-Pak™). Each was shipped FedEx® Priority Overnight.

### Leukocyte isolation by Ficoll gradient

Density gradient centrifugation was used to isolate leukocytes from a peripheral blood sample by spinning blood over Ficoll Paque Plus in a SepMate-50 (STEMCELL technologies, Inc., Canada) tubes following the manufacturer’s protocol. Isolated cells were resuspended in 1 ml of PBS and 1 × 10^6^ cells were aliquoted for cell sorting.

### Leukocyte isolation by whole blood lysis (RBC lysis)

Whole blood lysis was performed by mixing 1 ml of whole blood with 10 ml of erythrocyte hypotonic lysis buffer (161 μM NH_4_Cl in MilliQ water) and incubating for 5 min at room temperature. The blood samples were then spun and the supernatants were removed by vacuum aspiration. An additional 10 ml of erythrocyte hypotonic lysis buffer was added to each blood sample, incubated for 5 min at room temperature, and spun again. The cell pellets were washed three times with PBS, resuspended in 1 ml of PBS and 1 × 10^6^ cells were aliquoted for cell sorting.

### Leukocyte isolation by DNAse/collagenase digestion and Percoll gradient

Whole blood was processed in a modified brain mononuclear cell preparation described previously [21]. Briefly, peripheral blood samples were passed through an 18-gauge needle. Then, 200 μl of collagenase/dispase (10 mg/ml) and 600 μl DNAse (10 mg/ml) were added to each vacutainer and incubated at 37 °C for 1 h. Samples were then diluted to 14 ml with PBS and spun at 500 RCF for 5 min. The plasma layer was discarded by aspirating with a vacuum, and then 1 ml of the packed formed blood elements was mixed with 70% Percoll solution (3.1 ml isotonic Percoll mixed with 0.9 ml HBSS) and layered under a 30% Percoll solution (2.6 ml isotonic Percoll mixed with 5.4 ml RPMI). The Percoll gradient was centrifuged at 500 RCF for 20 min without brakes. The cells were harvested from the 30:70 interface using a transfer pipette. The cells were washed three times with PBS, resuspended in 1 ml of PBS, and 1 × 10^6^ cells were aliquoted for cell sorting.

### Leukocyte isolation by filtration and subsequent hypotonic lysis

LeukoLock filters were used with a modified protocol from the manufacturer’s instructions. The filters were sterilized by incubating the filters in 1.0 M NaOH and 2.0 M NaCl for 48 h at room temperature and were then washed by flushing twice with MilliQ water and twice with 70% ethanol. The filters were then vacuum dried and stored prior to use. Immediately prior to use the filters were flushed with 10 ml sterile PBS. After flushing, undiluted blood (6 mL) was pipetted directly from the vacutainer with a transfer pipette into the barrel of the syringe and allowed to pass through the filter by gravity. After the blood had passed through, the filters were rinsed by flushing with 3 ml of PBS. Leukocytes were harvested from the filter by connecting a female Luer coupler to the opposite end of the filter, and backflushing with 20 mL PBS into a new tube. The cell suspensions were pelleted at 300 RCF for 8 min and then resuspended in 5 mL Pharm Lyse buffer (BD) and incubated for 15 min. The cell suspensions were then washed twice with PBS, counted, and then resuspended in 1 ml of PBS and 1 × 10^6^ cells were aliquoted for cell sorting.

### Cell cover

When indicated, cells were lightly fixed in CellCover (Anacyte) after isolation but before staining for FACS by resuspending the cells in 1 ml ice-cold CellCover and incubating on ice for 10 min. The cells were spun at 300 RCF for 8 min and then stained for cell sorting. After staining, the cells were resuspended in 300 μl of ice-cold CellCover instead of azide-free FACS buffer and kept on ice before sorting.

### Isolation of leukocytes from intracerebral hemorrhage patients and healthy controls

Peripheral blood (8.5 mL) was collected from ICH patients and healthy controls in BD Vacutainer collection tubes with acid citrate dextran (Fisher Scientific) as an anti-coagulant and shipped overnight at 4 °C as described above. Leukocytes were isolated as described in the methods section titled “Leukocyte Isolation by Filtration and Subsequent Hypotonic Lysis”. Leukocytes were incubated in CellCover for 10 min on ice and then washed with HBSS. T cells were separated from other leukocytes by magnetic selection prior to FACS using an EasySep™ Human CD3 Positive Selection Kit II (Stem Cell) according to the manufacturer’s instructions and subsequently, the CD3^+^ and CD3^−^ fractions were stained individually. Briefly, cells were washed in ice-cold EasySep buffer (2% FBS, 1 mM EDTA in PBS) and subsequently stained with antibodies detailed in Additional file 1: Table S1 on ice for 15 min. Cells were incubated on ice prior to sorting on a BD FACS Aria II for CD8^+^ T cells and CD14^+^ monocytes.

### Fluorescence-activated cell sorting

Leukocyte populations were labeled with fluorescent conjugated antibodies and processed for fluorescence activation cell sorting (FACS). Antibodies used for each sorting panel and target cell population in this study are shown in Additional file 1: Table S1. Briefly: 1 × 10^6^ cells washed with 1 ml of PBS and centrifuged at 500 RCF for 5 min. The supernatants were aspirated, and the cell pellets were resuspended in 50ul human AB serum for 10 min to block Fc receptors. After Fc Block, 50 μl of the antibody cocktail was added to each sample and incubated for 20 min. The cells were washed with 1 ml PBS and centrifuged at 500 RCF for 5 min. The cells were then stained for dead cell exclusion by resuspending them in LIVE/DEAD Fixable Red Dead Cell Stain solution diluted 1:10,000 in PBS and incubated for 20 min. The samples were then washed with 1 ml of azide-free FACS buffer (2% BSA *w*/*v* and 1 mM EDTA in PBS) and spun at 500 RCF for 5 min. The supernatants were removed, and the cells were resuspended in 300 ul azide-free FACS buffer and kept on ice until cell sorting. All cell populations were isolated using fluorescent-activated cell sorting FACS Aria II (BD Biocsciences). The cells were sorted directly into 100 μl lysis buffer (RA1 spiked with 10 mM TCEP) and frozen at − 80 °C.

### Preparation of RNA-seq libraries

RNA was extracted using the NucleoSpin RNA XS Kit (Macherey-Nagel) according to the manufacturers instructions. cDNA synthesis and amplification were performed using SMARTer Ultra Low Input RNA V3 or V4 as indicated for Illumina Sequencing (Clontech) according to the manufacturers instructions. The input RNA was normalized prior to cDNA generation by diluting to ~ 1,000 cells per reaction. Paired-end sequencing libraries were prepared using the Nextera XT DNA sample Prep Kit (Illumina) according to the manufacturers instructions. Libraries were pooled in an equimolar ratio and sequenced on a NextSeq500 sequencer with 200 cycles per lane (Illumina).

### Transcriptional analysis

All samples were processed with STAR (v2.4.1d) and RSEM (v1.2.30). STAR was run on eight threads to align reads to a previously constructed transcriptome, with a reduced number of “spurious” junctions. A maximum number of 20 different alignments were allowed for each read. If this maximum was exceeded, the read was considered unmapped. A minimum overhang of 8 was permitted for unannotated junctions and a minimum overhang of 1 was permitted for annotated junctions. The maximum number of allowed mismatches per pair was 999, and the allowable intron length was between 10 and 1,000,000. The maximum genomic distance between mates was 1,000,000 and most of these settings used were the default according to the STAR manual provided by the developer (STAR --runThreadN 8 --runMode alignReads --genomeDir /home/Genomes/hg19_75_STAR/ --readFilesIn $read1 $read2 --outFilterType BySJout --outFilterMultimapNmax 20 --alignSJoverhangMin 8 --alignSJDBoverhangMin 1 --outFilterMismatchNmax 999 --alignIntronMin 10 --alignIntronMax 1000000 --alignMatesGapMax 1000000 --outFileNamePrefix STAR_output/${sampleprefix} --outSAMtype BAM SortedByCoordinate --quantMode TranscriptomeSAM). RSEM was run on paired-end reads, calculating 95% credibility intervals and posterior mean estimates (rsem-calculate-expression --paired-end --calc-ci -bam –p 8). FPKMs were log transformed post adding pseudocount of 1 and all down-stream analyses were performed on coding genes.

### Principal component analysis, clustering, and ssGSEA

Principal component analysis (PCA) was performed on interquartile-normalized data filtered for log_2_ (FPKM+ 1) > 0.1 using custom scripts in MATLAB (vR2015a) using the pca() function. Clustering was performed using Morpheus (hierarchical, one minus Pearson correlation) available through the Broad Institute or custom scripts in MATLAB. To enable global comparison across conditions to infer biological changes induced or masked by sample handling, single sample gene set enrichment (ssGSEA, v7) was performed [17] using the following settings: C2CPv5.1 gene sets, rank, weighting at 0.75, and minimum gene set size of 10. Significant gene sets were identified by paired t tests (with and without Benjamini-Hochberg FDR) of each condition to Ficoll isolated or fresh isolated, then merged and clustered hierarchically.

### Statistical analysis

Statistical analysis, including one-way ANOVA with Tukeys multiple comparisons test and students t test, was performed in Prism (v 7.0c). Statistical tests performed are indicated in the figure legends where data is presented.

## Additional files

Additional file 1: Supplemental Material contains the following data: **Figure S1**. Sorting strategy used to profile peripheral blood mononuclear cells (PBMCs) from blood of healthy donors across all conditions tested. Antibodies are listed in Table 1. **Figure S2**. Exon/intergenic ratios are plotted for each indicated condition for (**A**) monocytes, (**B**) T cells and (**C**) for filtration as compared to ficoll. Statistically significant comparisons are indicated and were calculated by one-way ANOVA with Tukey’s multiple comparisons test. **Figure S3.** Pairwise scatter plots of coding transcriptomes generated from monocytes for each indicated comparison. Regression lines and R^2^ values are shown on each plot for **(A**) ficoll, percoll and lysis processing conditions, and (**B**) ficoll, 4 °C for 1 day or 20 °C for 1 day conditions. **Figure S4.** Pairwise scatter plots of coding transcriptomes generated from CD8^+^ T cells for each indicated comparison. Regression lines and R^2^ values are shown on each plot for **(A**) ficoll, percoll and lysis processing conditions, and (**B**) ficoll, 4 °C for 1 day or 20 °C for 1 day conditions. **Figure S5**. ssGSEA results for ficoll and filter methods for isolation of PBMCs. Forest plots of top 15 significantly altered gene sets when PBMCs are isolated using filters for monocytes (**A**) and CD8^+^ T cells (**B**). **Figure S6**. Flow cytometry isolation scheme for sequencing data generated from cells isolated from intracerebral hemorrhage (ICH) and matched healthy donors (HD). **Figure S7**. Quality control metrics for sequencing data generated from cells isolated from intracerebral hemorrhage (ICH) and matched healthy donors (HD). (**A**) Exon/intergenic ratio for each indicated condition. No statistically significant differences were found when comparing healthy to ICH within each cell type by students t test. (**B**) Percent mapped reads for each indicated condition. No statistically significant differences were found when comparing healthy to ICH within each cell type by students t test for each percent metric plotted. **Table S1.** Antibodies used for cell sorting in this study. **Table S2**. Summary statistics performed by one-way ANOVA with Tukey’s multiple comparisons test for data shown in Fig. 2. (DOCX 3717 kb)

Additional file 2: **Table S3**. Quality control metrics for each library generated. Sample names, figure corresponding to data, cell type, and condition are indicated. (XLSX 65 kb)

Additional file 3: **Table S4**. ssGSEA results and significant comparisons. (XLSX 86 kb)

Additional file 4: **Table S5**. *P* values for each comparison of ssGSEA results for Fig. 5. Gene sets for which any comparison yielded a significant (*p* < 0.05) value is shown. (XLSX 52 kb)

## Abbreviations

CTLA-4: Cytotoxic-T-lymphocyte-associated protein 4
HD: Healthy donor
ICH: Intracerebral hemorrhage
NO1: Nitric oxide 1
p75 NTR: Neurotrophin receptor p75
PBMCs: Peripheral blood mononuclear cells
PCA: Principal component analysis
PCR: Polymerase chain reaction
PI3K AKT: Phosphatidylinositol-4,5-bisphosphate 3-kinase/protein kinase B
RBC: Red blood cell
RNA: Ribonucleic acid
ssGSEA: Single sample gene set enrichment analysis
TNFR1: TNF Receptor Superfamily Member 1A
VEGF: Vascular endothelial growth factor
