# Correction to: blood handling and leukocyte isolation methods impact the global transcriptome of immune cells

**DOI:** 10.1186/s12865-018-0273-9

**Published:** 2018-12-21

**Authors:** Brittany A. Goods, Jacqueline M. Vahey, Arthur F. Steinschneider, Michael H. Askenase, Lauren Sansing, J. Christopher Love

**Affiliations:** 1 Department of Biological Engineering, Koch Institute for Integrative Cancer Research at the Massachusetts Institute of Technology, Cambridge, MA 02139 USA; 2 Department of Electrical Engineering and Computer Science, Massachusetts Institute of Technology, Cambridge, MA 02139 USA; 3 Department of Neurology, Yale School of Medicine, New Haven, CT 06520 USA; 4 Department of Chemical Engineering, Koch Institute for Integrative Cancer Research at the Massachusetts Institute of Technology, Cambridge, MA 02139 USA; 5 The Broad Institute of the Massachusetts Institute of Technology and Harvard, Cambridge, MA 02142 USA

**Correction to: BMC Immunology (2018) 19:30**

10.1186/s12865-018-0268-6

It has been highlighted that the original article [1] contained a typesetting mistake in the middle name of Arthur F. Steinschneider. This was incorrectly captured as Arthur S. Steinschneider in the original article which has since been updated.
