# Supplementary material for: Blood handling and leukocyte isolation methods impact the global transcriptome of immune cells
Source: BMC Immunol. 2018 Oct 30;19:30. doi: 10.1186/s12865-018-0268-6 (PMC6208098; doi:10.1186/s12865-018-0268-6)
Supplement: Supplementary file 1 — Supplemental Material contains the following data: Figure S1. Sorting strategy used to profile peripheral blood mononuclear cells (PBMCs) from blood of healthy donors across all conditions tested. Antibodies are listed in Table 1. Figure S2. Exon/intergenic ratios are plotted for each indicated condition for (A) monocytes, (B) T cells and (C) for filtration as compared to ficoll. Statistically significant comparisons are indicated and were calculated by one-way ANOVA with Tukey’s multiple comparisons test. Figure S3. Pairwise scatter plots of coding transcriptomes generated from monocytes for each indicated comparison. Regression lines and R2 values are shown on each plot for (A) ficoll, percoll and lysis processing conditions, and (B) ficoll, 4 °C for 1 day or 20 °C for 1 day conditions. Figure S4. Pairwise scatter plots of coding transcriptomes generated from CD8+ T cells for each indicated comparison. Regression lines and R2 values are shown on each plot for (A) ficoll, percoll and lysis processing conditions, and (B) ficoll, 4 °C for 1 day or 20 °C for 1 day conditions. Figure S5. ssGSEA results for ficoll and filter methods for isolation of PBMCs. Forest plots of top 15 significantly altered gene sets when PBMCs are isolated using filters for monocytes (A) and CD8+ T cells (B). Figure S6. Flow cytometry isolation scheme for sequencing data generated from cells isolated from intracerebral hemorrhage (ICH) and matched healthy donors (HD). Figure S7. Quality control metrics for sequencing data generated from cells isolated from intracerebral hemorrhage (ICH) and matched healthy donors (HD). (A) Exon/intergenic ratio for each indicated condition. No statistically significant differences were found when comparing healthy to ICH within each cell type by students t test. (B) Percent mapped reads for each indicated condition. No statistically significant differences were found when comparing healthy to ICH within each cell type by students t test for each percent metri [file 12865_2018_268_MOESM1_ESM.docx]

**Supplemental Material for:**

**Title:** Blood handling and leukocyte isolation methods impact the global transcriptome of immune cells

**Authors:** Brittany Goods^1,^*, Jacqueline M. Vahey^1^†, Arthur S. Steinschneider^2^†, Michael H. Askenase^2^, Lauren Sansing^2^‡, J. Christopher Love^1,3,4^‡*

^1^Department of Biological Engineering, Koch Institute for Integrative Cancer Research at MIT, Cambridge, Massachusetts 02139, USA.

^2^Department of Neurology, Yale School of Medicine, New Haven, Connecticut 06520, USA.

^3^Department of Chemical Engineering, Koch Institute for Integrative Cancer Research at MIT Cambridge, Massachusetts 02139, USA.

^4^The Broad Institute of MIT and Harvard, Cambridge, MA 02142, USA.

† These authors contributed equally to this work.

‡ These authors contributed equally to this work.

*Correspondence to: [clove@mit.edu](mailto:clove@mit.edu), [bagoods@mit.edu](mailto:bagoods@mit.edu)


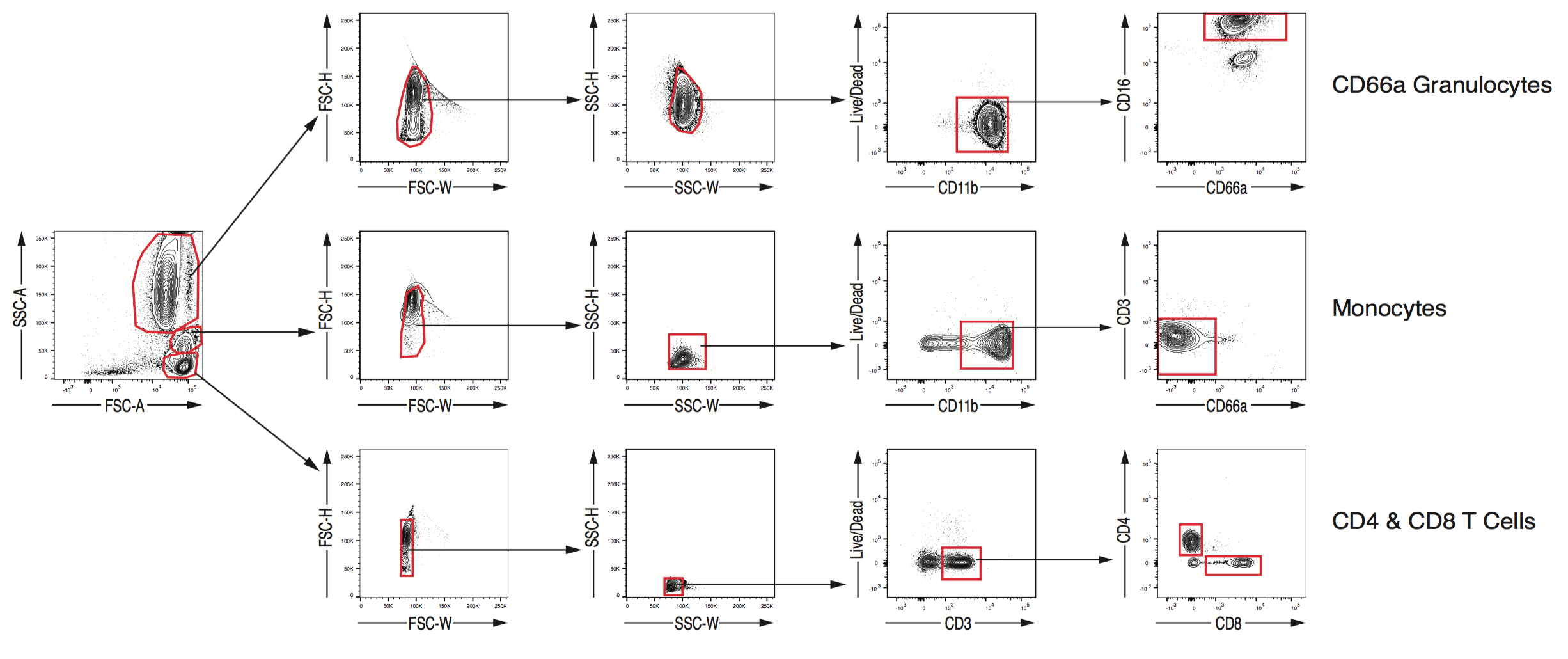


**Supplemental Figure 1**. Sorting strategy used to profile peripheral blood mononuclear cells (PBMCs) from blood of healthy donors across all conditions tested. Antibodies are listed in Table 1.


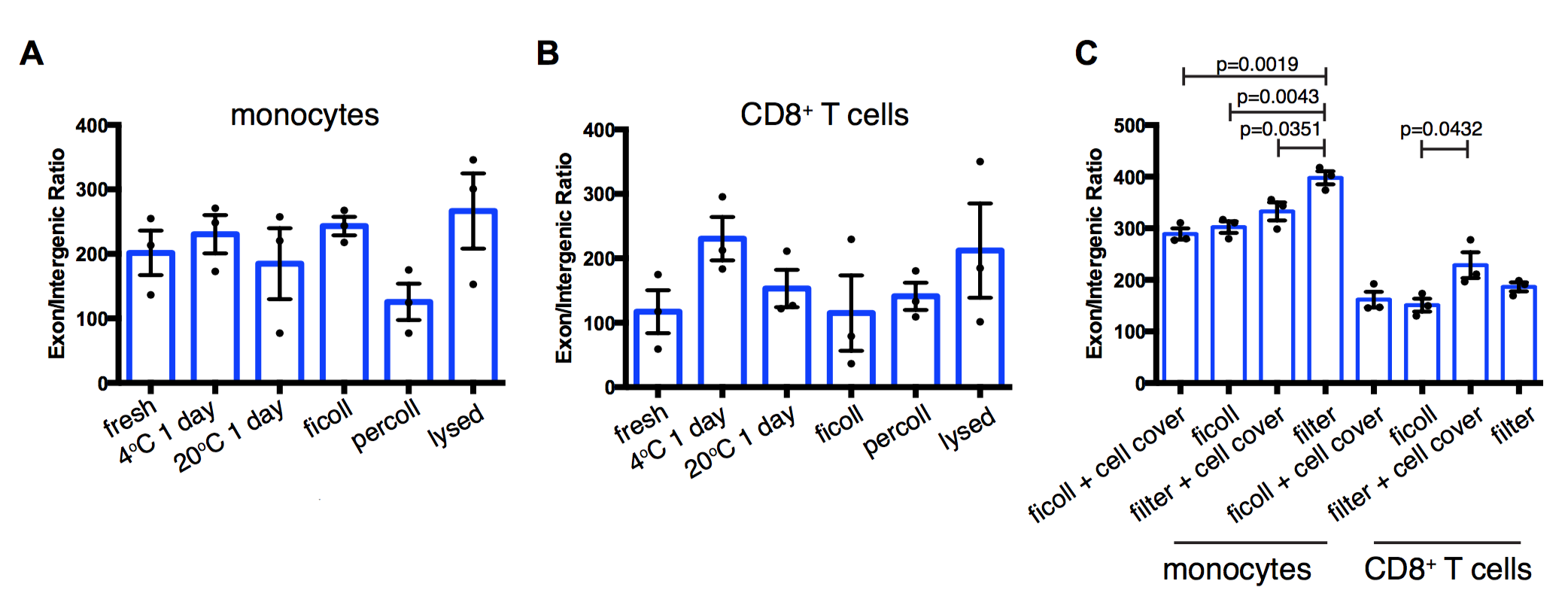


**Supplemental Figure 2**. Exon/intergenic ratios are plotted for each indicated condition for (**A**) monocytes, (**B**) T cells and (**C**) for filtration as compared to ficoll. Statistically significant comparisons are indicated and were calculated by one-way ANOVA with Tukey’s multiple comparisons test.


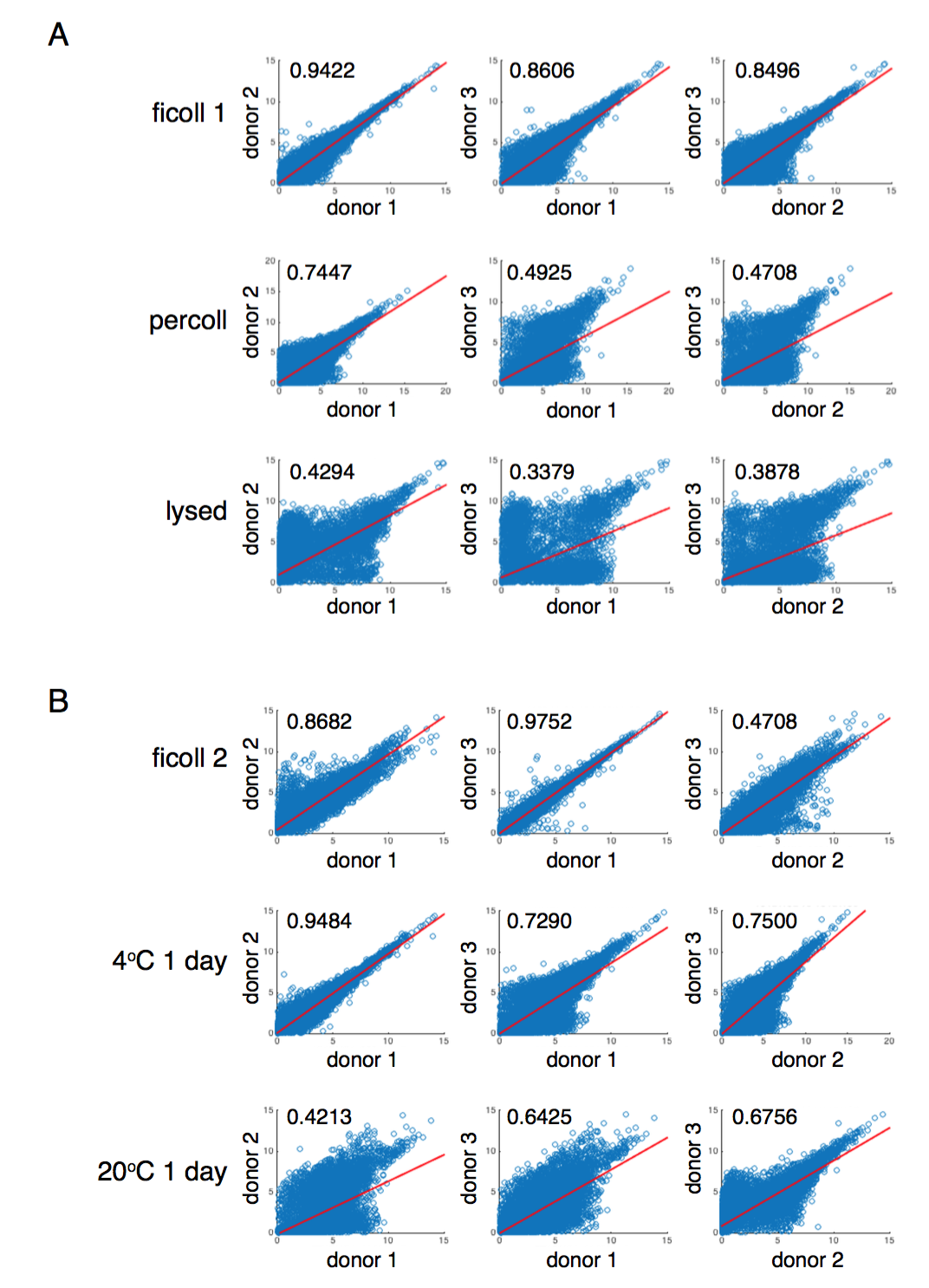


**Supplemental Figure 3. Pairwise scatter plots of coding transcriptomes generated from monocytes for each indicated comparison.** Regression lines and R^2^ values are shown on each plot for **(A**) ficoll, percoll and lysis processing conditions, and (**B**) ficoll, 4°C for 1 day or 20°C for 1 day conditions.


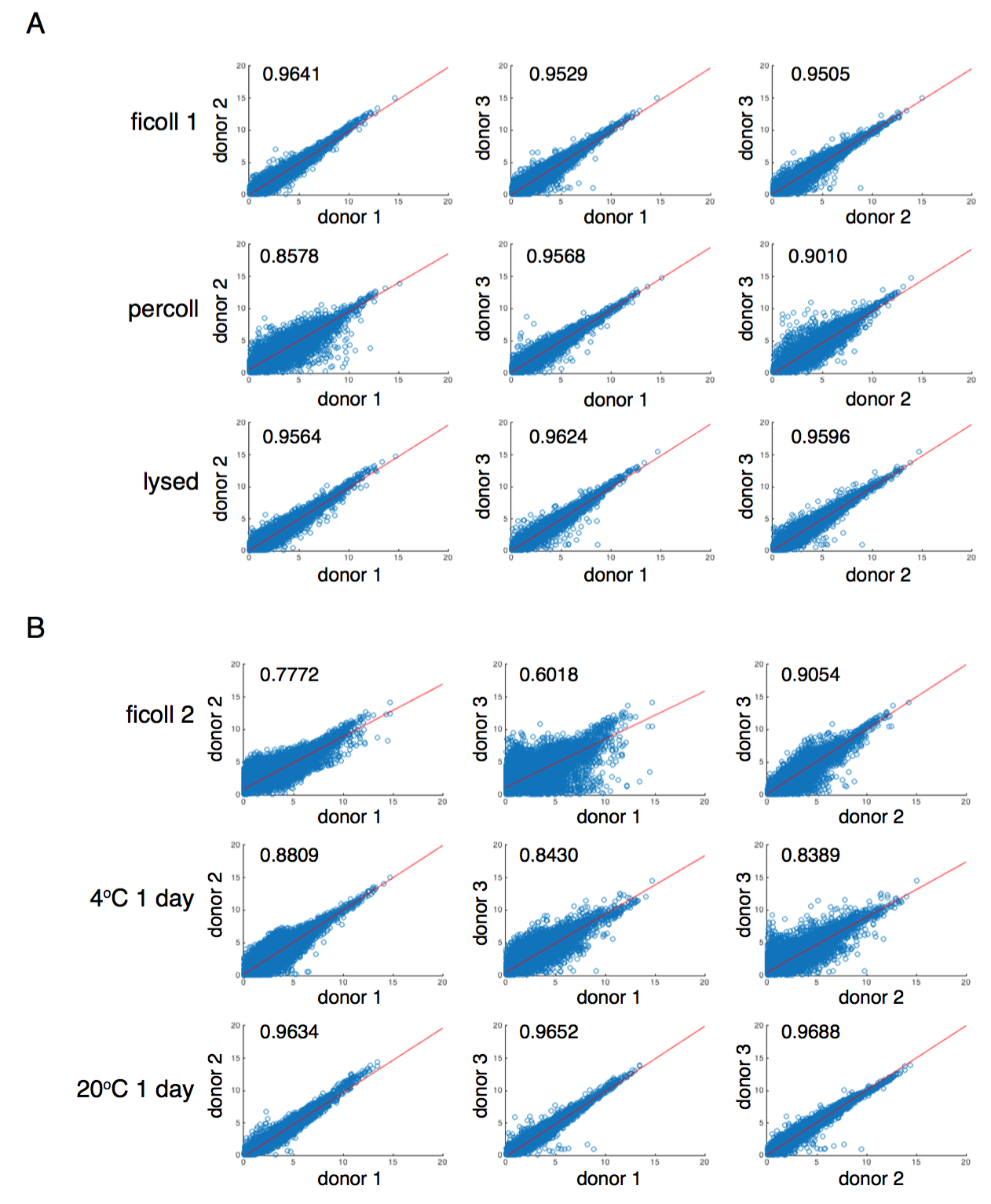


**Supplemental Fig 4. Pairwise scatter plots of coding transcriptomes generated from CD8^+^ T cells for each indicated comparison.** Regression lines and R^2^ values are shown on each plot for **(A**) ficoll, percoll and lysis processing conditions, and (**B**) ficoll, 4°C for 1 day or 20°C for 1 day conditions.


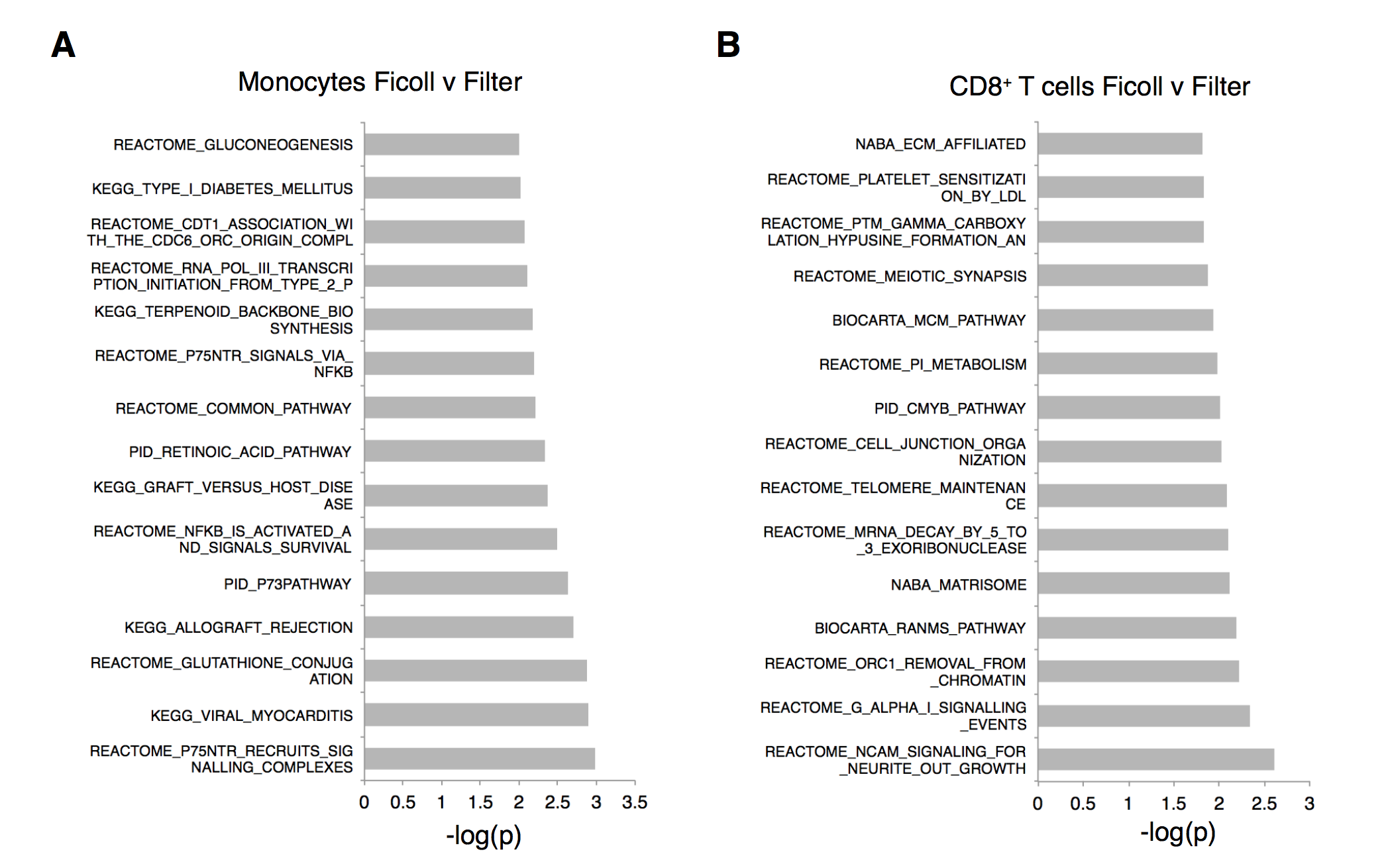


**Supplemental Figure 5.** **ssGSEA results for ficoll and filter methods for isolation of PBMCs.** Forest plots of top 15 significantly altered gene sets when PBMCs are isolated using filters for monocytes (**A**) and CD8^+^ T cells (**B**).


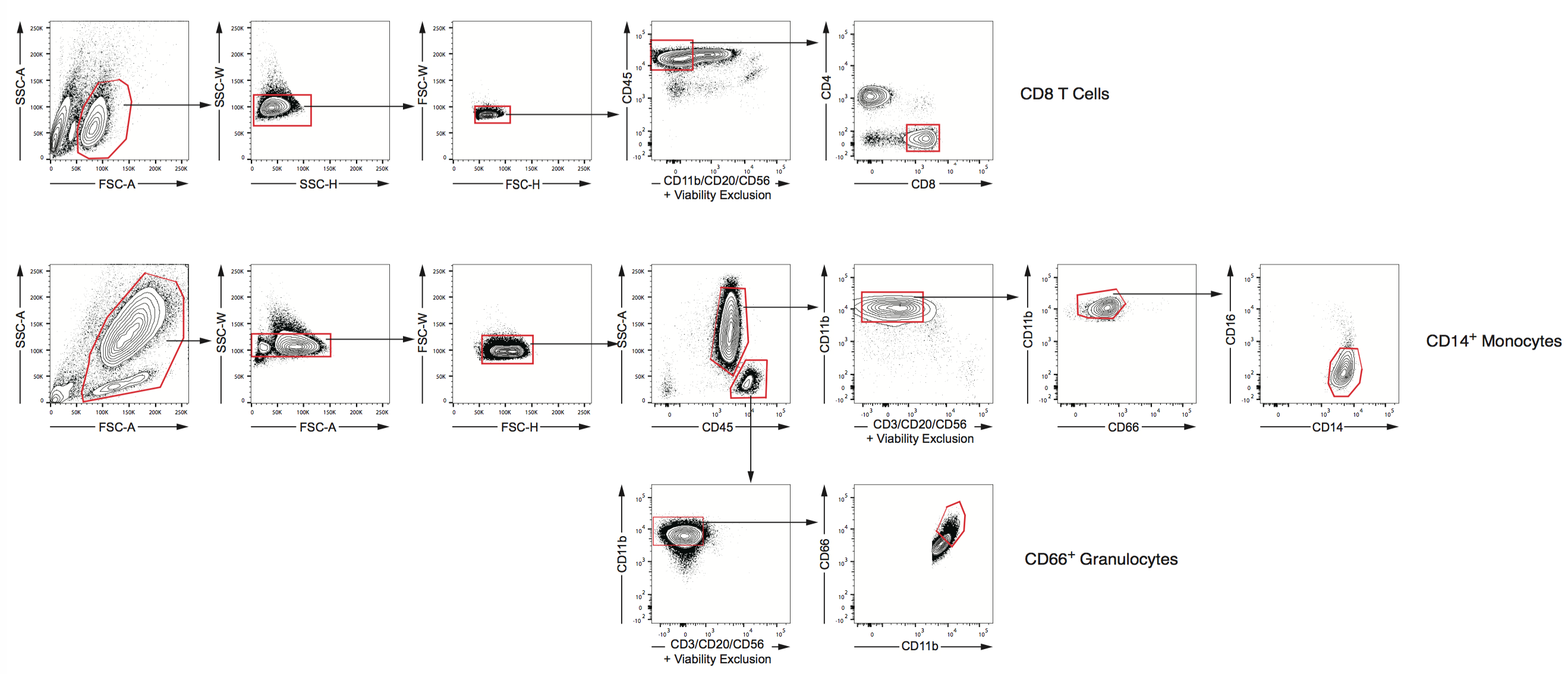


**Supplemental Figure 6**. Flow cytometry isolation scheme for sequencing data generated from cells isolated from intracerebral hemorrhage (ICH) and matched healthy donors (HD).


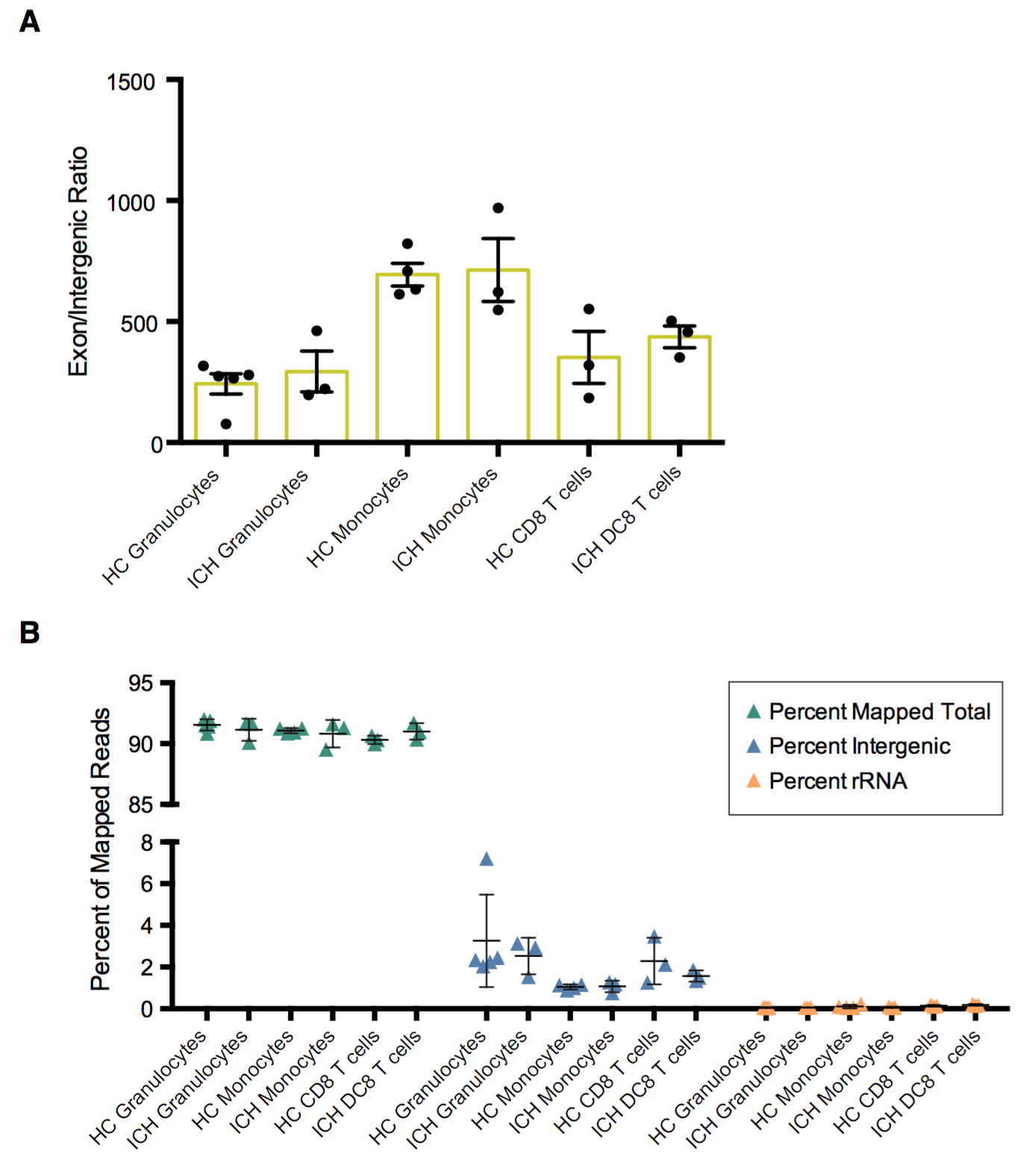


**Supplemental Figure 7**. Quality control metrics for sequencing data generated from cells isolated from intracerebral hemorrhage (ICH) and matched healthy donors (HD). (**A**) Exon/intergenic ratio for each indicated condition. No statistically significant differences were found when comparing healthy to ICH within each cell type by students t test. (**B**) Percent mapped reads for each indicated condition. No statistically significant differences were found when comparing healthy to ICH within each cell type by students t test for each percent metric plotted.

**Supplemental Table 1. Antibodies used for cell sorting in this study.**

| **Supplemental Table 1: Antibodies used for the cell sorting** | | | | |
| --- | --- | --- | --- | --- |
| 1. **Sort panel I** | | | | |
| **Marker** | **Label** | **Clone** | **Supplier** | **Catalog #** |
| CD3 | APC/H7 | SK7 | BD | 560176 |
| CD4 | V450 | RPA-T4 | BD | 560345 |
| CD8 | V500 | RPA-T8 | BD | 560774 |
| CD11b | FITC | ICRF44 | eBioscience | 11-0118-42 |
| CD16 | PE/Cy7 | 3G8 | BD | 557744 |
| CD25 | PE | M-A251 | BD | 555432 |
| CD45 | PerCP/Cy5.5 | H130 | Tonbo | 65-0459-T100 |
| CD66a | PE | B1.1 | BD | 564105 |
| CD127 | AF 647 | HIL-7R-M21 | BD | 558598 |
| Live/Dead | Fixable red dye |  | Life Technologies | L34972 |
| 1. **Sort panel II – T cells** | | | | |
| **Marker** | **Label** | **Clone** | **Supplier** | **Catalog #** |
| CD2 | APC/H7 | RPA-2.10 | BD | 562638 |
| CD4 | violetFluor 450 | RPA-T4 | Tonbo | 75-0049-T100 |
| CD8 | PE/Cy7 | RPA-T8 | Tonbo | 60-0088-T100 |
| CD11b | Biotin | M1/70 | Tonbo | 30-0112-U500 |
| CD20 | Biotin | 2H7 | Biolegend | 302350 |
| CD25 | PE | M-A251 | BD | 555432 |
| CD45 | FITC | HI30 | Tonbo | 35-0459-T100 |
| CD56 | Biotin | HCD56 | Biolegend | 318320 |
| CD127 | APC | R34-34 | Tonbo | 20-1278-T100 |
| Streptavidin | PE-CF594 |  | BD | 562318 |
| Live/Dead | Fixable red dye |  | Life Technologies | L34972 |
| 1. **Sort panel II – Innate cells** | | | | |
| **Marker** | **Label** | **Clone** | **Supplier** | **Catalog #** |
| CD2 | Biotin | RPA-2.10 | Biolegend | 300204 |
| CD11b | FITC | ICRF44 | Tonbo | 35-0118-T100 |
| CD14 | APC/Cy7 | M5E2 | Biolegend | 301820 |
| CD16 | v450 | 3G8 | BD | 560474 |
| CD20 | Biotin | 2H7 | Biolegend | 302350 |
| CD45 | PE | HI30 | Tonbo | 50-0459-T100 |
| CD56 | Biotin | HCD56 | Biolegend | 318320 |
| CD66a/c/e | PE/Cy7 | ASL-32 | Biolegend | 342310 |
| Streptavidin | BV510 |  | Biolegend | 405234 |
| Live/Dead | Fixable red dye |  | Life Technologies | L34972 |

**Supplemental Table 2**. Summary statistics performed by one-way ANOVA with Tukey’s multiple comparisons test for data shown in Figure 2.

| **Figure** | **Cell Type** | **Tukey's multiple comparisons test** | **Mean Diff.** | **95.00% CI of diff.** | **Significant?** | **Summary** | **Adjusted P Value** |
| --- | --- | --- | --- | --- | --- | --- | --- |
| **2A** | CD4 T cells | Freshly Isolated vs. 1 Day 20C | 14.31 | -1.799 to 30.42 | No | ns | 0.0767 |
|  | CD4 T cells | Freshly Isolated vs. 1 Day 4C | 20.44 | 4.336 to 36.55 | Yes | * | 0.0188 |
|  | CD4 T cells | 1 Day 20C vs. 1 Day 4C | 6.134 | -9.974 to 22.24 | No | ns | 0.5118 |
|  | CD8 T cells | Freshly Isolated vs. 1 Day 20C | 6.237 | -3.844 to 16.32 | No | ns | 0.2193 |
|  | CD8 T cells | Freshly Isolated vs. 1 Day 4C | 7.647 | -2.434 to 17.73 | No | ns | 0.1272 |
|  | CD8 T cells | 1 Day 20C vs. 1 Day 4C | 1.41 | -8.671 to 11.49 | No | ns | 0.905 |
|  | Monocytes | Freshly Isolated vs. 1 Day 20C | 2.31 | -5.792 to 10.41 | No | ns | 0.6743 |
|  | Monocytes | Freshly Isolated vs. 1 Day 4C | 3.249 | -4.852 to 11.35 | No | ns | 0.4798 |
|  | Monocytes | 1 Day 20C vs. 1 Day 4C | 0.9396 | -7.162 to 9.041 | No | ns | 0.9333 |
|  | Granulocytes | Freshly Isolated vs. 1 Day 20C | -3.483 | -9.523 to 2.556 | No | ns | 0.2573 |
|  | Granulocytes | Freshly Isolated vs. 1 Day 4C | -14.86 | -20.89 to -8.816 | Yes | *** | 0.0007 |
|  | Granulocytes | 1 Day 20C vs. 1 Day 4C | -11.37 | -17.41 to -5.333 | Yes | ** | 0.0028 |
| **2B** | CD4 T cells | Ficoll Gradient vs. Whole Blood Lysis | 21.83 | 8.283 to 35.37 | Yes | ** | 0.0062 |
|  | CD4 T cells | Ficoll Gradient vs. Collagenase + Percoll Gradient | 26.84 | 13.3 to 40.39 | Yes | ** | 0.0022 |
|  | CD4 T cells | Whole Blood Lysis vs. Collagenase + Percoll Gradient | 5.019 | -8.523 to 18.56 | No | ns | 0.5283 |
|  | CD8 T cells | Ficoll Gradient vs. Whole Blood Lysis | 10.18 | 1.301 to 19.06 | Yes | * | 0.0291 |
|  | CD8 T cells | Ficoll Gradient vs. Collagenase + Percoll Gradient | 10.79 | 1.914 to 19.68 | Yes | * | 0.0227 |
|  | CD8 T cells | Whole Blood Lysis vs. Collagenase + Percoll Gradient | 0.6132 | -8.268 to 9.494 | No | ns | 0.9757 |
|  | Monocytes | Ficoll Gradient vs. Whole Blood Lysis | 9.179 | -4.263 to 22.62 | No | ns | 0.1711 |
|  | Monocytes | Ficoll Gradient vs. Collagenase + Percoll Gradient | -11.97 | -25.41 to 1.471 | No | ns | 0.076 |
|  | Monocytes | Whole Blood Lysis vs. Collagenase + Percoll Gradient | -21.15 | -34.59 to -7.708 | Yes | ** | 0.007 |
|  | Granulocytes | Ficoll Gradient vs. Whole Blood Lysis | -38.12 | -54.85 to -21.39 | Yes | ** | 0.001 |
|  | Granulocytes | Ficoll Gradient vs. Collagenase + Percoll Gradient | -17.54 | -34.27 to -0.8039 | Yes | * | 0.0418 |
|  | Granulocytes | Whole Blood Lysis vs. Collagenase + Percoll Gradient | 20.58 | 3.853 to 37.32 | Yes | * | 0.0216 |
| **2C** | CD4 T cells | Ficoll vs. Ficoll + Cellcover | -2.658 | -14.36 to 9.04 | No | ns | 0.8834 |
|  | CD4 T cells | Ficoll vs. Whole Blood Filtration | 10.22 | -1.474 to 21.92 | No | ns | 0.0884 |
|  | CD4 T cells | Ficoll vs. Whole Blood Filtration + Cellcover | 10.08 | -1.622 to 21.77 | No | ns | 0.0936 |
|  | CD4 T cells | Ficoll + Cellcover vs. Whole Blood Filtration | 12.88 | 1.184 to 24.58 | Yes | * | 0.0318 |
|  | CD4 T cells | Ficoll + Cellcover vs. Whole Blood Filtration + Cellcover | 12.73 | 1.036 to 24.43 | Yes | * | 0.0336 |
|  | CD4 T cells | Whole Blood Filtration vs. Whole Blood Filtration + Cellcover | -0.1481 | -11.85 to 11.55 | No | ns | >0.9999 |
|  | CD8 T cells | Ficoll vs. Ficoll + Cellcover | -0.7315 | -7.549 to 6.086 | No | ns | 0.985 |
|  | CD8 T cells | Ficoll vs. Whole Blood Filtration | -0.4832 | -7.301 to 6.334 | No | ns | 0.9955 |
|  | CD8 T cells | Ficoll vs. Whole Blood Filtration + Cellcover | -0.3196 | -7.137 to 6.498 | No | ns | 0.9987 |
|  | CD8 T cells | Ficoll + Cellcover vs. Whole Blood Filtration | 0.2484 | -6.569 to 7.066 | No | ns | 0.9994 |
|  | CD8 T cells | Ficoll + Cellcover vs. Whole Blood Filtration + Cellcover | 0.412 | -6.406 to 7.229 | No | ns | 0.9972 |
|  | CD8 T cells | Whole Blood Filtration vs. Whole Blood Filtration + Cellcover | 0.1636 | -6.654 to 6.981 | No | ns | 0.9998 |
|  | Monocytes | Ficoll vs. Ficoll + Cellcover | 4.604 | -3.839 to 13.05 | No | ns | 0.3625 |
|  | Monocytes | Ficoll vs. Whole Blood Filtration | 12.23 | 3.782 to 20.67 | Yes | ** | 0.0073 |
|  | Monocytes | Ficoll vs. Whole Blood Filtration + Cellcover | 13.91 | 5.47 to 22.36 | Yes | ** | 0.0033 |
|  | Monocytes | Ficoll + Cellcover vs. Whole Blood Filtration | 7.621 | -0.8225 to 16.06 | No | ns | 0.0777 |
|  | Monocytes | Ficoll + Cellcover vs. Whole Blood Filtration + Cellcover | 9.309 | 0.8657 to 17.75 | Yes | * | 0.0316 |
|  | Monocytes | Whole Blood Filtration vs. Whole Blood Filtration + Cellcover | 1.688 | -6.755 to 10.13 | No | ns | 0.916 |
|  | Granulocytes | Ficoll vs. Ficoll + Cellcover | 0.1653 | -15.45 to 15.78 | No | ns | >0.9999 |
|  | Granulocytes | Ficoll vs. Whole Blood Filtration | -39.91 | -55.52 to -24.29 | Yes | *** | 0.0002 |
|  | Granulocytes | Ficoll vs. Whole Blood Filtration + Cellcover | -30.85 | -46.46 to -15.23 | Yes | ** | 0.001 |
|  | Granulocytes | Ficoll + Cellcover vs. Whole Blood Filtration | -40.07 | -55.68 to -24.46 | Yes | *** | 0.0002 |
|  | Granulocytes | Ficoll + Cellcover vs. Whole Blood Filtration + Cellcover | -31.01 | -46.63 to -15.4 | Yes | *** | 0.001 |
|  | Granulocytes | Whole Blood Filtration vs. Whole Blood Filtration + Cellcover | 9.059 | -6.554 to 24.67 | No | ns | 0.3162 |
